# Effects of Intercropped Insectary Plants (Sweet Alyssum, Coriander, and White Mustard) on Elemental Composition and Antioxidant Levels in Broad Bean Plants

**DOI:** 10.3390/molecules29246031

**Published:** 2024-12-21

**Authors:** Janina Gospodarek, Gedyon Tamiru, Aleksandra Nadgórska-Socha, Marta Kandziora-Ciupa, Iwona B. Paśmionka

**Affiliations:** 1 Department of Microbiology and Biomonitoring, University of Agriculture, al. A. Mickiewicza 21, 31-120 Krakow, Poland; tamiru.menagedyon.sd@student.urk.edu.pl (G.T.); iwona.pasmionka@urk.edu.pl (I.B.P.); 2 Department of Ecology, University of Silesia, Bankowa 9, 40-007 Katowice, Poland; aleksandra.nadgorska-socha@us.edu.pl (A.N.-S.); marta.kandziora-ciupa@us.edu.pl (M.K.-C.)

**Keywords:** biological protection against pests, macro- and microelements in soil and broad bean, soil enzymes, antioxidant response

## Abstract

Insectary plants, such as sweet alyssum, coriander, and white mustard, are well known for their traits that attract beneficial insects, allowing them to protect crops from pests. The aim of the study was to analyze the compounds that are important in the antioxidant response, such as malondialdehyde, ascorbic acid, proline, total phenolics, and total flavonoids, as well as the content of elements, including macroelements (K, Mg, Na, Ca, P, and S) and heavy metals (Cd, Cu, Zn, Pb, Ni, Mn, and Fe) in broad bean plants. These plants were grown in field conditions as the main protected plant alongside a mixture of three insectary plants at different proportions of the individual components. The soil was analyzed in terms of the above-mentioned elements, as well as in terms of its enzymatic activity (arylsulfatase, *β*-glucosidase, dehydrogenase, FDA (fluorescein diacetate), and acid phosphatase). The introduction of insectary plant mixtures did not cause major changes in the content of the elements in the soil. The changes in the content of elements in broad bean leaves depended on the type of element and the proportion of individual components in the companion plant mixture. However, a general trend of increasing macronutrient content was observed, influenced by the presence of companion plants. All types of companion plant mixtures used enhanced the activity of FDA, while the mixture with 50% sweet alyssum additionally caused an increase in arylsulfatase activity (more than 2 fold). The companion plants improved the physiological condition of the protected plant, which was reflected in the reduced content of proline and total flavonoids. Considering the response of the protected plant to the proposed intercropped plant mixtures and their effect on broad bean growth, it appears that the most suitable mixtures are those with an equal share of all three plant species or a mixture with a predominance of sweet alyssum.

## 1. Introduction

Introducing plants with properties that attract beneficial insects to increase the predation and parasitism of crop pests is one of the widely studied ways to protect plants from pests [1,2]. It is a method that relies on enhancing the natural resistance of the environment by increasing both the abundance and species diversity of beneficial entomofauna, resulting in the creation of more sustainable trophic relationships in agrocenoses and, thus, providing a chance to avoid herbivore gradations. To date, many plant species have been studied for their suitability for the above-mentioned purpose. Among these plants, three species are noteworthy: sweet alyssum (*Lobularia maritima* L.) (SA), white mustard *(Sinapis alba* L.) (MU), and coriander (*Coriandrum sativum* L.) (CO) [3,4]. These plants have been shown to have a positive effect on predators such as Syrphidae (MU, SA, CO), Coccinellidae (MU, SA, CO), Carabidae (MU, SA), Anthocoridae (SA), Staphylinidae (SA, CO), Araneae (SA, CO), and Chrysopidae (CO), as well as parasitoids (MU, CO) [3,4,5,6,7,8,9,10]. Furthermore, the pollen of these plants in many cases showed positive effects on the developmental parameters of beneficial entomofauna, such as fertility (MU—Syrphidae), longevity (MU, SA, CO—parasitoids), and egg-laying duration (CO—Syrphidae) [3]. Each of these plants has advantages from the point of view of their use as companion plants, as well as some limitations. The advantages of SA as a companion plant include its small size, which reduces competition with the main plant, the short period from sowing to flowering, and the long flowering period. However, a disadvantage is its susceptibility to being fed upon by certain pests (flea beetles), which excludes it from being used as a companion plant for other host plants for these pests [5,11]. MU blooms quickly after sowing and produces a significant amount of pollen and nectar, but its disadvantages are the fact that it grows quickly and reaches a large size, which can adversely affect the growth of the main plant. CO, on the other hand, does not reach as large a size as MU and flowers for a long time but blooms relatively late.

The use of the above three plant species in a mixture with the right proportions to minimize disadvantages and maximize the opportunity to use the attributes of these plants can benefit the protection of the main plant from pests more than either plant used separately. The effects of these plants on pest incidence, beneficial insects, and crop yield are fairly well recognized [6,7,8,9,10,11,12,13,14]. However, the available literature lacks any information on the effects of such insectary plant mixtures used as companion plants on the chemical composition of the protected main plant and its physiological response, as well as the enzymatic activity of the soil.

Adjacent plants in mixed crops can interact with each other through competition for nutrients, water, or light. However, plant root excretions modify the solubility and bioavailability of nutrients and can thus influence their uptake and the subsequent transfer to above-ground parts by plants growing alongside. This phenomenon can lead to an increase in plant nutrient acquisition due to complementarity (a decrease in competition between plants in the mix due to spatial and temporal variation in resource use) [15]. Moreover, additional plants can influence the enzymatic activity of the soil and the physiological response of the protected plant [16,17].

Soil enzymes as the biomolecules responsible for the circulation of elements in the soil are also useful soil quality indicators [18]. Among those of key importance are arylsulfatase, which is responsible for making sulfur available to plants, *β*-glucosidase, which is active in the final stage of cellulose decomposition [19], and dehydrogenase, which is responsible for the degradation of organic matter [18]. Fluorescein diacetate (FDA) hydrolysis, on the other hand, is known to be an accurate and simple method for measuring soil microbial activity and is also correlated positively with soil quality [20], while phosphatases catalyze the hydrolysis of organic phosphorous forms and can increase P availability to plants [21].

Physiological parameters, such as malondialdehyde (MDA) or proline content, can indicate the level of stress a plant is under. MDA is a breakdown product of polyunsaturated fatty acid hydroperoxides that are formed in reactions with reactive oxygen species (ROS), which in turn, are early signals of the plant defense response to stress factors. Proline is a proteinogenic amino acid. It is considered to be a unique defensive substance in plants [22,23,24]. Its increase has been observed under the influence of factors such as soil and air drought, salinization, high and low temperatures, heavy metals, phosphorous starvation, ultraviolet, and phytopathogens, among others. The mechanisms of proline action are multifaceted. It serves as a chelator of metals; stabilizes cellular structures under stress; protects against the denaturation of different proteins; inactivates ROS; and activates antioxidant enzymes [24]. The total phenolics (TP) and total flavonoids (TF) in the plant exhibit redox properties and act as reducing agents and hydrogen donors. Together with ascorbic acid, they are plant components with a wide spectrum of positive health effects [25]. In addition, TP and TF in the plant activate defense mechanisms under pest attack [26].

The broad bean (*Vicia faba* L.), due to its valuable composition (high protein content, B and C vitamins, soluble fiber, and mineral salts), is used both as an edible crop for humans and as animal feed and is cultivated practically worldwide today [27,28]. Furthermore, its ability to fix atmospheric nitrogen by symbiosis with *Rhizobium* bacteria enhances and sustains soil productivity [29]. The literature also highlights its role in increasing the diversification of the wild flora, wild fauna, and soil microbes that have an indirect beneficial effect on the entire agroecosystem.

The aim of this study was to analyze (a) the compounds that are important in the antioxidant response, such as MDA, ascorbic acid, proline, TP, and TF, as well as the content of elements, including macroelements (K, Mg, Na, Ca, P, and S) and heavy metals (Cd, Cu, Zn, Pb, Ni, Mn, and Fe), in broad bean plants. These plants were grown in field conditions as the main protected plant alongside a mixture of three insectary plant species—SA, CO, and MU—at different proportions of the individual components of the mixture (equal proportions of all species—mcs; the dominance of SA—mcSA; the dominance of CO—mCOs; and dominance of MU—MUcs); (b) the effect of such arranged pest management scenarios for the broad bean on the content of the above-mentioned macroelements and heavy metals in the soil and soil enzymatic activity (arylsulfatase, *β*-glucosidase, dehydrogenase, FDA, and acid phosphatase); and (c) the impact of the proposed mixtures on the morphological parameters of broad beans such as length of stem, number of stems per plant, number of composite leaves per stem, and mass of aboveground part of plant.

We assumed the following hypotheses:

1. the introduction of companion plants will induce changes in the content of individual elements in the soil and consecutively in the protected plant, and these changes will depend on the type of element and the proportion of individual companion plants in the mixture.
2. companion plants will enhance the activity of soil enzymes and the strength of the effect will depend on the composition of the mixture.
3. companion plants, by protecting the main plant from pest attack, will improve the physiological condition of the protected plant, which will be reflected in the level of parameters such as MDA, ascorbic acid, proline, TP, and TF.

The proposed choice of plants for the mixture was based on the results of previous studies on the effects of these plants introduced individually as intercrops in broad beans for pest control [7,30,31,32,33,34,35]. MU could provide an early start in attracting beneficial entomofauna since it blooms earliest and is visible from a considerable distance. SA, while retaining the previously mentioned beneficial effects regarding insects, does not pose as much of a competitor to the main plant due to its relatively small size. CO, on the other hand, would maintain a long-lasting attractive effect, due to its long flowering period.

## 2. Results

The soil parameters, as well as the granulometric composition of the soil taken from the experiment at the same time as sampling for analysis of soil elements and enzymes, did not show large differences depending on the protection scenario adopted (Tables S1 and S2).

### 2.1. Elements Content in the Soil and Soil Enzyme Activity

The mixture of insectary plants with CO dominance (mCOs) resulted in a significant increase in the content of K, Mg, Na, Ca, P, and S, as well as Cd, Cu, Ni, and Mn in the soil compared to the control (Table 1, Table S3). In addition, treatment with chemical protection resulted in significantly higher Na, P, and Pb content. There was no significant effect of the mixtures used on soil Zn and Fe content.

**Figure 1:**
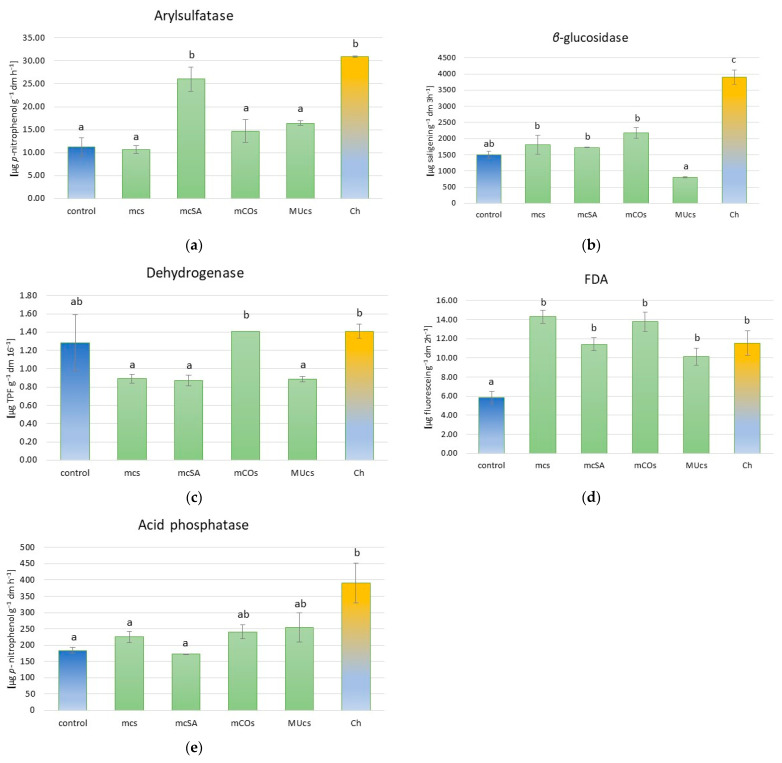
Soil enzyme activity levels. (**a**) Arylsulfatase; (**b**) *β*-glucosidase; (**c**) dehydrogenase; (**d**) FDA; (**e**) acid phosphatase. For description of treatments, see Table 1. Different letters above bars indicate statistically significant differences (*p* ≤ 0.05, Tukey’s test).

The increased proportion of SA (mcSA) in the mix, as well as chemical protection, resulted in an increase in arylsulfatase activity (Figure 1a, Table S4). *β*-glucosidase activity was significantly higher in the treatment with chemical protection compared to the control and all treatments with mixtures of insectary plants, with the enzyme showing the lowest activity in the treatment with the highest proportion of MU (Figure 1b). The broad bean protection treatments used did not significantly affect dehydrogenase activity relative to the control, but significant variation was noted between treatments, with a significantly higher activity by these enzymes in the treatment with increased CO (mCOs) and in the treatment with chemical protection compared to the other treatments with accompanying plants (Figure 1c). All modes of broad bean protection contributed to a significant increase in FDA activity, while a significant increase in acid phosphatase activity was recorded only for chemical protection (Figure 1d,e).

### 2.2. Element Contents in Broad Bean

The introduction of mixtures of insectary plants, regardless of the proportion of individual components, resulted in higher K, Ca, S, and Fe contents in broad bean tissues compared to the control (Table 2, Table S5). With S and Fe, the greatest increase was recorded when the proportion of individual plants in the mixture was equal (mcs, two and three times, respectively), followed by an increased proportion of MU (MUcs). In the case of Mg, the introduction of companion plants also resulted in an increase in the concentration of this element in the broad bean, excluding treatment with a 50% share of CO (mCOs). A significant increase in Na content was recorded due to the introduction of broad bean cultivation mixtures with a 50% share of CO (mCOs, over 2 times) and MU (MUcs, by 80%) and under the influence of chemical protection (by 60%). In turn, the P content increased significantly due to the introduction of a mixture with an equal proportion of plants (mcs) and a predominance of MU (MUcs).

There was no effect of any of the protection modes on the content of Pb, Ni, and Mn in the broad bean. Increased Cd content was noted in the treatment with a 50% share of SA (mcSA), while an increased Cu content was noted in the treatment with a predominance of MU (MUcs). The zinc content was the highest in the tissues of the broad beans grown with the mixture with an equal share of individual plants (mcs, twice as high as in the control), followed by MUcs and mcSA.

### 2.3. Physiological Parameters in Broad Bean

**Figure 2:**
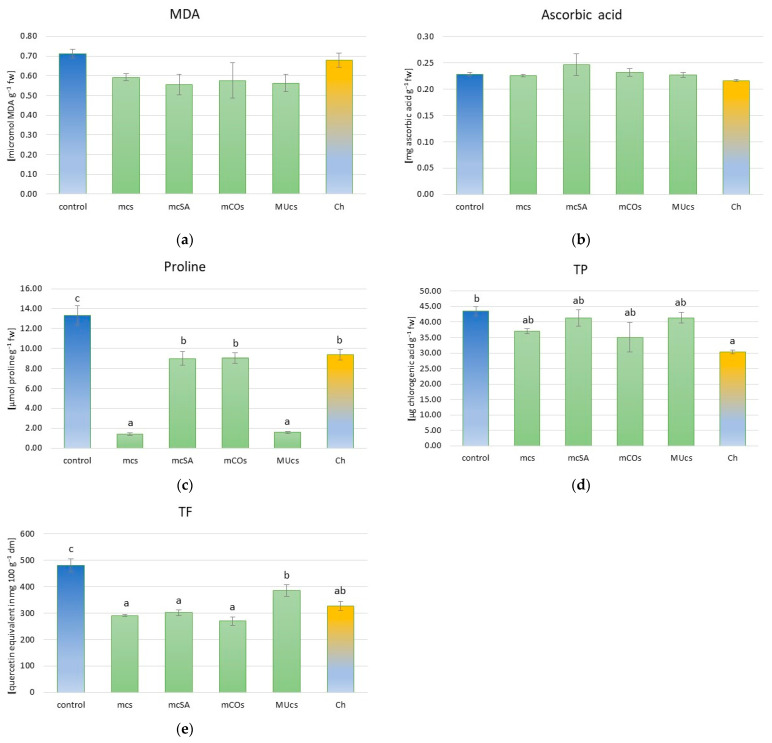
Physiological parameters in broad bean leaves. (**a**) malondialdehyde (MDA) content; (**b**) ascorbic acid content; (**c**) proline content; (**d**) total phenolics (TP) content; (**e**) total flavonoids (TF) content. For description of treatments, see Table 1. Different letters above bars indicate statistically significant differences (*p* ≤ 0.05, Tukey’s test). Where letters are not present, no significant differences were found.

There was no significant effect of the tested protection methods on the content of MDA and ascorbic acid in broad bean leaves (Table S6, Figure 2a,b). However, significant differences in proline content were noted between the treatments (Table S6, Figure 2c). The highest content of this compound was noted in the control plants. Significantly lower content was noted in the leaves of broad beans from the mSA, mCOs, and chemically protected treatments (a decrease of about 45%). In contrast, in broad beans from the mcs and MUcs treatments, the proline content was about 9- and 8-fold lower, respectively. The TP content did not differ significantly between the protection methods, except for chemical protection. In the latter treatment, the TP content was the lowest and was significantly lower than in the control (Figure 2d). The TF content was the highest in the control plants. All applied protection methods significantly reduced the contents of these compounds in the following order: control > MUcs > Ch > mcSA > mcs > mCOs (Figure 2e).

### 2.4. Growth Parameters of Broad Bean

Broad beans obtained the lowest values of growth parameters in the unprotected control, both in 2023 and 2024 (Table 3). In 2023 the stems of broad beans were longest in the treatment with the highest proportion of MU (MUcs), followed by treatment with an equal share of companion plants in the mixture (mcs) and with a predominance of SA (mcSA). All types of protection positively influenced this parameter (Table 3, Table S7). Both chemical protection and the use of companion plant mixtures with an equal proportion of plants (mcs) and the dominance of SA (mcSA) resulted in a significantly greater branching of the broad bean plants in 2023. The number of leaves per stem was not affected by the treatments. The weight of the aboveground part of the plant was significantly (positively) affected only by chemical protection and the presence of companion plants in equal proportions (mcs). Broad bean plants reached a significantly smaller size in the 2024 season. Plant height (length of stem) and number of leaves per stem in this season were significantly positively affected only by chemical protection. In contrast, broad beans developed the most shoots when grown with a mixture of companion plants with equal proportions, (mcs) while the weight of the aboveground part was highest when a mixture with the highest proportion of SA was used.

### 2.5. Relationships Between Soil and Broad Bean Chemical Composition, Soil Enzymes Activity, and Physiological and Growth Parameters of Broad Bean

**Figure 3:**
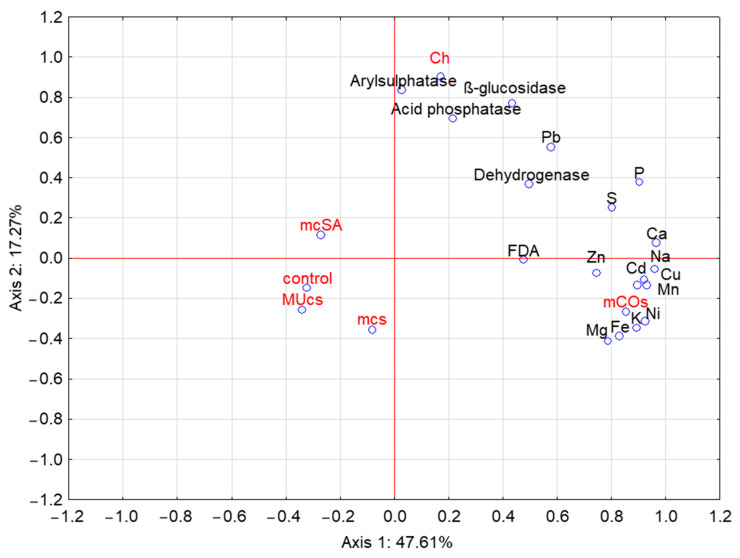
Principal component analysis of element levels and enzymatic activity of the soil. Symbols as in Table 1 and Figure 1.

A principal component analysis (PCA) of the element levels and enzymatic activity of the soil for the different protection variants allowed for the identification of four principal components, two of which explained 64.88% of the variation in the analyzed samples (Figure 3). The elements studied were positively correlated with the first ordination axis. The same was true for the soil enzymes, although this correlation was weaker. In contrast, the soil enzymes (with the exception of FDA) and Pb, S, and P were clearly positively correlated with the second ordination axis. Elements such as Mg, Fe, K, and Ni, on the other hand, were negatively correlated with this axis. PCA also confirmed the positive effect of the CO-dominant mixture on the contents of Mg, Fe, K, and Ni in particular, and of chemical protection on the activity of the enzymes, such as arylsulfatase, *β*-glucosidase, and acid phosphatase.

**Figure 4:**
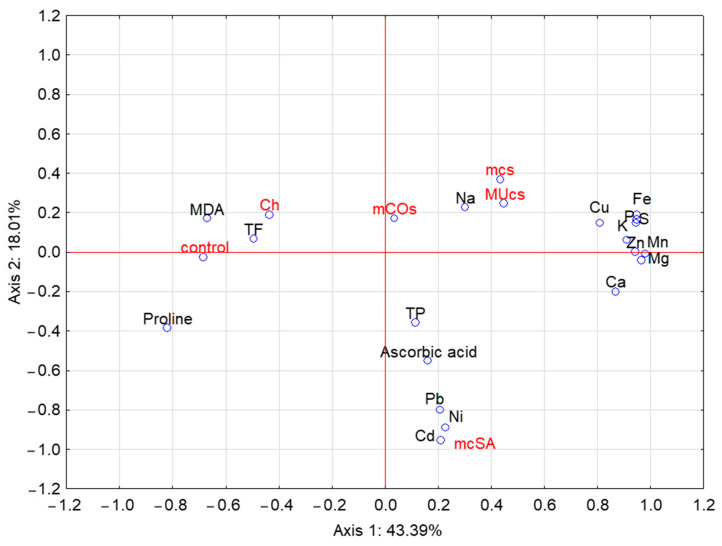
Principal component analysis of element levels and physiological parameters of the broad bean. Symbols as in Table 1 and Figure 2.

A PCA of the element levels and physiological parameters of the broad bean allowed for three main components to be distinguished. Two of these components together explained 61.40% of the variation (Figure 4). The contents of Ca, Mg, Zn, Mn, K, S, P, Cu, and Fe were positively correlated with axis I, while the contents of MDA, proline, and TF were negatively correlated with this axis. A negative correlation with axis II was found for the contents of Cd, Ni, Pb, and ascorbic acid. The latter parameters can also be linked to the use of an SA-dominant mixture.

**Figure 5:**
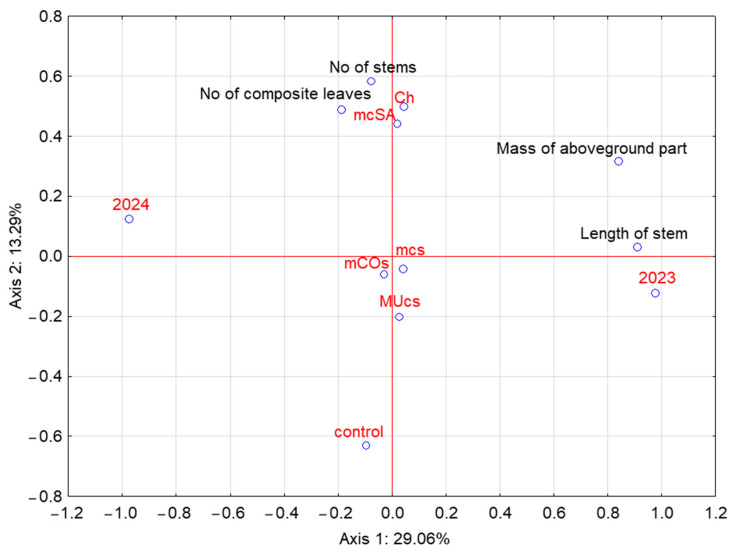
Principal component analysis of growth parameters of the broad bean. Symbols as in Table 1.

A PCA of the growth parameters of the broad bean allowed for the extraction of three principal components, two of which explained a total of 42.35% of the variation (Figure 5). The first axis was mainly positively correlated with parameters such as the mass of the aboveground part of the broad bean plants and the length of the stems. In contrast, a positive correlation with the second axis was found for the number of stems and the number of composite leaves. The analysis also confirmed a clear difference between the two research seasons.

## 3. Discussion

A previous study on the effects of MU, CO, and SA used separately as co-crops for the broad bean showed that each of these crops has some advantages that are relevant to pest management, as well as some disadvantages. MU sown in the inter-rows of broad beans helped increase the abundance of eggs and larvae of hoverflies and improve the ratio of predators (ladybugs) to prey (aphids) [7]. In addition, there was an increase in the length of the primary root and the above-ground part of seedlings and in the number of lateral roots of broad beans under the influence of MU proximity [30]. However, the higher seed yield of broad beans was obtained only with simultaneously increased inter-row spacing [7]. CO as a companion plant for broad beans reduced the degree of leaf damage by the pea leaf weevil, *Sitona* spp. [31] and infection by *Botrytis fabae* Sardiña, *Uromyces viciae-fabae* (Pers.) J. Schröt, and *Botrytis cinerea* Pers. [32]. However, it also contributed to an increase in the degree of seed colonization by pathogenic and saprotrophic fungi [33] and, due to its size, reduced yield [34]. On the other hand, SA in broad bean cultivation reduced the abundance of the black bean aphid *Aphis fabae* Scop., contributed to an increase in the abundance of hoverflies and ladybugs, and did not compete with the broad beans, even when maintaining the standard spacing for broad beans grown alone (i.e., 50 cm) [35]. Given these observations, it seemed reasonable to test a mixture of these plants, with different proportions of each component, to potentially maximize their desired effect and reduce the disadvantages.

### 3.1. Content of Elements in the Soil and Soil Enzyme Activity

The different protection options tested in this experiment did not affect the HNO_3_-extractable fraction of elements in the soil in most cases. The exception was the soil from the treatment in which CO dominated the companion plant mix. In this case, the elemental content was 18% higher for K, 16% higher for Mg, 40% higher for Na, 19% higher for Ca, 36% higher for P, 13% higher for S, 23% higher for Cd, 43% higher for Cu, 19% higher for Ni, and 13% higher for Mn, respectively, compared to the soil from the control treatment. Thus, the hypothesis we formulated regarding the possible influence of mixtures of insectary plants on the elemental content of the soil was only partially confirmed. Higher contents of tested elements than in the unprotected control were also recorded in the variant with chemical protection, but they concerned only Na (12% higher), Ca (8% higher), P (29% higher), and Pb (33% higher). There is no information in the available literature on the effect of growing CO as a companion crop on the elemental content of the soil. The observed differences may be due to the complex action of the root secretions of the plants growing together, as well as the depth and distribution of the root systems of the plants in the mixture in this specific cultivation scenario (i.e., a CO-dominant mixture).

We also hypothesized that mixtures of companion plants would enhance the activity of soil enzymes. This hypothesis was confirmed for all treatments in the case of FDA, while for the other soil enzymes, the effect of the neighborhood of the plant mixtures was generally not statistically significant with respect to the control (except for arylsulfatase in the treatment mSA). Arylsulfatase activity increased in the soil where broad beans grew alone and were protected with synthetic pesticides (more than 2.5 fold) and in an SA-dominant mixture (more than 2-fold). This enzyme is responsible for the metabolism of sulfur compounds, including increasing its availability to plants as nutrition [36]. A significant increase in the activity of this enzyme was found in soil where the crop rotation included soil organic matter enrichment through the introduction of intercrops in winter wheat (mustard) and spring barley (clover) [37] and under the influence of a wheat–corn intercrop [38]. In doing so, a positive correlation of this enzyme with total S, N, and C_org_ was found. In our experiment, however, the total S content in Ch and mcSA sites was at a similar level to that in the control, while the N_t_ and C_org_ contents were even lower (C_org_ in mcSA only). The difference may be due to the length of time that the factors under investigation had been operating. The soil samples in the above-mentioned experiments were taken after many years of efforts to increase soil organic matter content, whereas in the present experiment, the differentiating factor (companion plants) only had a chance to have an effect for a period of about three months—from sowing at the end of March to sampling in mid-June. Moreover, according to some authors [39], there is no direct relationship between the activity of this enzyme and the content of nitrogen supplied to the soil in the form of mineral salts, which agrees with the present results. Higher arylsulfatase activity was found in coffee intercropped with *Brachiaria decumbens* [16,40]. The authors explained the increased activity of this enzyme (as well as *β*-glucosidase) by the constantly falling litter on the soil surface, lower amplitude of the soil temperature, and better soil moisture, which directly affects soil microorganisms. Favorable moisture conditions may be a partial explanation for the increased arylsulfatase activity in the mcSA treatment, as SA, being a small-sized plant, does not compete strongly for water. Moreover, it contributes to soil shading through its creeping plant shape. Mixtures with increased amounts of CO and MU due to the higher water requirements of these plants may have resulted in reduced moisture in these treatments. In contrast, in the control, where the growth of the broad bean was markedly reduced compared to the chemically protected treatment, a reduction in interplant shading could also have resulted in poorer soil moisture and, thus, reduced soil enzyme activity. These factors (shade and soil moisture) may also be responsible for the higher *β*-glucosidase activity in the Ch treatment recorded in our experiment. This is also supported by the finding of the lowest activity of this enzyme in the treatment with the highest proportion of MU in the mixture (MUcs). MU, due to its intensive growth, has the highest water requirement of the three plants tested in this experiment. As for the effect of synthetic pesticides on soil enzymes, their response can vary according to soil type and pesticide type, causing enhancement or inhibition of arylsulfatase and dehydrogenase activity [41]. In the present experiment, the duration of the interaction of companion plants with the soil was relatively short, but significant changes in some soil enzyme activities were nevertheless observed. Similarly, a study of a system of 35 cover crops (including intercropping) interacting for a period of 3 months (from the end of February to the end of May) also showed significant effects on soil enzyme activity. As a result, it was possible to find plant species (bean, white lupin, *Crotalaria spectabilis*, common vetch, corn, and *Eleusine coracana*) in stand-alone crops and plant groups (white oat + rye + field pea + pivoting turnip, oat IPR Afrodite + black oat + fodder radish, buckwheat + millet, *Crotalaria juncea* + fodder radish, common oat + millet, common oat + millet + fodder radish, and black oat + millet + fodder radish) in mixed crops, resulting in higher arylsulfatase and *β*-glucosidase activity [42]. As can be seen, both plant species in stand-alone crops and in mixtures belong to different systematic families, confirming the wide variation in soil enzymatic response to the presence of different plant species.

Dehydrogenase activity in our experiment did not change significantly under the analyzed treatments with respect to the control, although higher activity was recorded in the treatment with predominant CO in the mixture (mCOs) and in the chemically protected site than in the other treatments with companion plants. Similarly, there was no effect of intercropping yam–alfalfa and yam–clover on the activity of this enzyme compared to yam monoculture [43], nor was there a short-term (after one cropping period) effect of soybean intercropping with cassava [44]. However, other studies have detected a positive effect of intercropping practices on dehydrogenase activity [45]. Particularly emphasized is the favorable role of legumes. In a pear orchard, dehydrogenase activity responded positively to any legume-based intercropping system [46], similar to the effect of the introduction of soybean as an intercrop in maize [47]. This may partly explain our observation of the highest activity of this enzyme in the Ch treatment, where broad bean plants had the strongest growth. In addition, soil dehydrogenase activity under low doses of insecticides can even increase, as has been demonstrated for monocrotophos, quinalphos, and cypermethrin applied separately to soil (although with mixtures of these insecticides, the response varied depending on the type of mixture and dose) [18]. In these studies, the increase in dehydrogenase activity may have been due to an increase in the activity of the microorganisms responsible for the degradation of the pesticides delivered to the soil.

The FDA-hydrolyzing activity increased almost 2 fold under the influence of all tested protection methods in the present experiment. Most studies confirm such a positive effect of intercropping practices on the mentioned parameter. Intercropping prairie cordgrass and kura clover caused an increase in FDA enzyme activity compared to monoculture prairie cordgrass fertilized with different nitrogen (N) rates of granular urea (five treatments: 0 N, 75 N, 150 N, and 225 N) [48]; *Pennisetum glaucum* + *Crotalaria spectabilis* used as cover crops in soybean increased FDA activity [49]; Soil enzyme activity, including FDA, was 20–34% higher when intercropping potato with two legumes, namely lima bean (*Phaseolus lunatus*) and dolichos (*Lablab purpureus*), than when growing potatoes alone [50]. However, growing corn with different species of tropical grasses did not result in any change in FDA activity compared to growing corn alone [51].

The activity of acid phosphatase increased exclusively under protection with synthetic pesticides. The reason for this seems to be the proven positive effect of legumes (such as lupin and faba bean) on the activity of this enzyme in the rhizosphere [52]. Phosphatases catalyze the hydrolysis of organic P forms and can increase the availability of P to plants. Legumes in particular are known as a group of plants that exude high amounts of phosphatases [21,53]. This could explain the recorded highest activity of this enzyme in the Ch treatment, where there were no companion plants and the broad bean plants were in good condition—not attacked by pests and diseases. This is also confirmed by the P content of the soil from this treatment, which was higher than in the unprotected control and in most of the sites with companion plants (except for mCOs). According to the literature, the effect of intercropping on the activity of this enzyme is generally positive. The intercropping of capsicum–maize resulted in an increase in acid phosphatase activities compared to homogeneous crops of capsicum and maize [54], as did maize intercropping with soybean [55]. In our experiment, the activity of this enzyme in the companion plant treatments was only slightly higher (differences not significant) than in the control treatment (except for mcSA, where it was slightly lower).

### 3.2. Element Contents in Broad Bean

The current research indicates the potential for improved nutrient acquisition in plant mixtures, due to root–architecture complementarity and differences in requirements between species and, thus, an increase in the uptake of several soil nutrients [56]. However, antagonisms that result in reduced element concentrations in tissues of plants growing in mixtures compared to sole crops are also reported [57]. The observed higher contents of HNO_3_-extractable fraction elements in the soil with mCOs treatment did not correspond to the content of these elements in the above-ground parts of broad beans. Extraction with HNO_3_ can detect up to several times higher metal contents than with other reagents. Potentially plant-available contents may vary for different elements and represent, for example, from 1–3% for Cu and Pb to 40% for Zn compared to the HNO_3_-extractable fraction [58]. However, extraction with HNO_3_ makes it possible to predict what might happen when soil conditions change (changes in pH, moisture content, and plant species) and, therefore, what amount of an element could potentially be released and become available to plants.

In general, most of the analyzed macronutrients showed significantly higher contents in the tissues of broad beans from treatments with companion plants than from the unprotected control. The predominance of MU in the mixture resulted in the highest contents of K, Mg, and P. The predominance of CO favored an increase in Na content. The predominance of SA resulted in the highest Ca content, while S reached the highest concentration in the treatment with equal proportions of all three plants in the mixture. Similarly, studies on crucifer–legume cover crops found higher nutrient uptake in legume shoots in species mixtures than in sole crops, indicating the compatibility and complementarity of crucifer and legume mixtures in the use of soil nutrients [59]. However, the authors also found negative competition for some elements (Cu and Fe), indicating the need to consider the specific conditions encountered when using a particular mixture. So far, there are only a few reports of antagonism in nutrient acquisition in plant mixtures. Peanuts in a mixture with maize had a lower Ca concentration [60]. Also, growing cucumber with green garlic resulted in lower Mg content [61]. There may be more such examples of competition for nutrients between plants in mixtures, but they have not yet been identified.

The K and P content of the broad bean increased by 70% with MUcs treatment compared to the control. As previously reported [52], MU shows significant potential to mobilize P from FePO_4_ in the soil. The total P content of corn biomass increased by 1.1 fold in corn–MU intercrops. Plants in the Brassicaceae family can cause changes in soil pH and exude significant amounts of carboxylates [62], which can contribute to the mobilization of certain elements, e.g., P [63]. The soil reaction in the Brassicaceae-dominated treatments in our experiment (mcSA and MUcs) was, indeed, slightly lower than in all other protected treatments. However, there are also reports that intercropping had no effect on P uptake by the main crop, although the companion plants did secrete carboxylates and alter the soil pH in the rhizosphere [64,65]. As for the effect of intercropping on potassium content, data on the effect of crucifers are scarce. The content of K (35.9 mmol/kg) in sugar beet grown together with MU was similar to the control (34.9 mmol/kg) [66]. The K content of legume shoots was not significantly different depending on whether they were grown as the sole crop or in a mixture with crucifers [59].

Cultivating broad beans with SA at different row spacings (50 cm, 65 cm, and 80 cm) resulted in a lower content of Mg and P in broad beans, while the content of Ca and K has increased in some cases in relation to the monoculture [57]. Similarly, another study [59] found an increase in Ca content in legumes when grown in mixtures with crucifers compared to sole legumes. In the SA-predominant plant mixture in the present experiment, significant increases in K (by 37%), Mg (by 52%), Ca (by 42%), and S (by 62%) were found, which partly agrees with the data of the authors mentioned above. Moreover, the elevated content of arylsulfatase in the soil interacts with the high (but not the highest) sulfur content of the broad bean from the mcSA treatment. As mentioned above, this is the enzyme responsible for, among other things, increasing the availability of this nutrient to plants.

The intercropping pattern of one row of CO and two rows of onion (*Allium cepa* L.) gave higher values of onion phosphorus (0.43 g/bulb in year one and 0.45 g/bulb in year two) compared to the sole onion crop in both seasons (0.35 g/bulb and 0.39 g/bulb in years one and two, respectively) [67]. However, intercropping tomato with CO did not affect the macronutrients (in g/kg of leaves as follows: N: 119.9, P: 13.2, K: 108.2, Ca: 85.7, Mg: 22.4, and S: 15.6) concentrations in the tomato leaf in comparison with monoculture (N: 118.1 g/kg, P: 15.1 g/kg, K: 104.4 g/kg, Ca: 75.6 g/kg, Mg: 19.5 g/kg, and S: 16.8 g/kg) [68]. In our experiment, the highest proportion of CO in the mixture resulted in higher content of K (by 48%), Na (over 2-fold), Ca (by 39%), and S (by 43%) in comparison to the control, while the Mg and P contents were not affected. So, the mechanism of nutrient acquisition can vary considerably among plant species [59].

The insectary plant mixtures we tested did not significantly affect the Pb, Ni, and Mn contents of broad bean leaves, while the Fe content increased under their influence. Some of the tested mixtures also contributed to an increase in Zn, Cu, and Cd. The results of other authors showed that the content of microelements and heavy metals in broad bean growing together with SA either did not vary much (Ba, Sr, and Cd) or increased (Fe, Ni, and Al) compared to the broad bean monoculture [57]. However, intercropping tomato with CO did not affect the micronutrients (in g/kg of leaves as follows: Bo: 0.4, Cu: 0.6, Fe: 0.9, Mn: 0.1, and Zn: 0.13) concentration in the tomato leaf in comparison with the monoculture (Bo: 0.3 g/kg, Cu: 0.7 g/kg, Fe: 0.7 g/kg, Mn: 0.09 g/kg, and Zn: 0.12 g/kg) [68]. A reduction in Cu and Fe content was found in legumes grown in a mixture with crucifers [59], whereas in the present experiment, growing broad beans with insectary plants did not affect the Cu content in the main plant tissues in most treatments (except MUcs, when it was 46% higher) while it caused an increase in Fe content (in all treatments with companion plants). The increase in Fe content in plant tissues as a result of intercropping practices is generally explained by the effect of these plants on increasing soil acidity, which increases the availability of some nutrients in the soil [69]. However, the analysis of soil pH at the individual sites in this experiment does not support this explanation. According to some data, the increase in Fe uptake may be the result of an increase in the expression of genes responsible for this process in intercropped plants compared to monoculture [70]. However, this requires further research. Moreover, as the study shows, even within the same family (Fabaceae), there were differences in Fe content depending on the species. In vetches and crimson clover, the content of this element was lower in the mixtures with crucifers. On the other hand, in Egyptian clover, soybean, and faba bean, the Fe content was higher when these plants were grown in a mixture with crucifers [59]. An analysis of cadmium and lead uptake of kenaf (*Hibiscus cannabinus* L.) and soybean (*Glycine max* L.) under intercropping in mining soil, showed that the Cd content increased in kenaf leaves, while the Pb content decreased in both the kenaf leaves and the soybean compared to a monoculture [71]. Our experiment showed no significant effect of intercropping on the Pb content, while Cd was only detected in broad bean tissues from the mcSA treatment.

### 3.3. Physiological Parameters in Broad Bean

The introduction of plant mixtures into the inter-rows did not significantly affect the MDA and ascorbic acid content of broad bean leaves. The MDA content in plants is correlated with the degree of membrane lipid peroxidation [72], which is a consequence of plant stresses. The available literature mostly indicates a beneficial effect of intercropping practices on plant physiological parameters, manifested by a reduction in MDA content in plants. The MDA content in eggplant intercropped with garlic (relay intercropping) was significantly lower than in an eggplant monoculture [73]. The authors highlighted that this indicates less damage from environmental factors in intercropping and, thus, better plant health. Similarly, the introduction of wheat into watermelon cultivation to reduce *Fusarium* wilt resulted in lower MDA content in watermelon roots [74]. The intercropping of guar, kochia, and *Sesbania* reduced guar leaf MDA content compared to a guar monocropping system [75]. A proso millet–mung bean intercropping system reduced the MDA in proso millet [76], while the intercropping of kenaf and soybean in mining soil reduced MDA levels in both these crops [71]. However, MDA levels were found to be increased in the ‘Summer Black’ grapevine under intercropping with four *Solanum* plants: *Solanum nigrum* var. *humile*, *Solanum diphyllum*, *Solanum nigrum*, and *Solanum alatum* [77]. Intercropping strawberries and broad beans did not significantly affect the ascorbic acid content of the fruit [78], while the ascorbic acid content of cabbage intercropped with different herbs varied significantly depending on the research season, as well as depending on the companion plant, reaching the highest values in one season of monoculture and in another by intercropping with thyme [79]. In contrast, the introduction of basil and cabbage plants into the tomato crop resulted in a lower content of ascorbic acid (44% lower) and polyphenols (11.1% lower) in the pulp compared to a cropping system based on the application of cow manure and manual weed control [17]. The authors explained this observation by the fact that the system with intercropping reduced the need for protective barriers against environmental stress.

The proline content in plants is postulated to be one of the first plant responses to stress, and its accumulation may be enhanced by biomass reduction [80]. This would explain the highest levels of this compound being observed in the control plants, which had the lowest biomass of the aboveground parts. Also, the stress caused by pests in this treatment was the highest. All the protection methods used significantly reduced the proline content compared to the control, with this effect being particularly pronounced in the mcs and MUcs treatments (more than six times lower proline levels than in the control). There are no data in the available literature on the effect of MU, CO, or SA used as companion plants on the proline content of the main plant. In contrast, data on the effect of intercropping practices on the proline content of legumes are diverse. Intercropping of winter faba bean and winter wheat significantly increased proline content, but only in one of the two faba bean genotypes tested (S_062) [80]. In contrast, in a study on legume–non-legume intercropping combined with the biofertilizers chickpea (*Cicer arietinum* L.) and dragon’s head (*Dracocephalum* L.), monocrops without fertilization had the highest proline levels, which is in agreement with the results of our study [81].

The protection methods used did not significantly affect the TP content, except in the treatment protected with synthetic pesticides, where its level was about 40% lower than in the control. This may be explained by the reduction in stress caused by pest feeding, especially the black bean aphid, in the chemically protected plots. Under pest-feeding stress, the TP content generally increases. This phenomenon was recorded, for example, in the case of feeding by the aphid *Rhopalosiphum padi* L. on wheat [26] or in the leaves of *Thuja orientalis* in response to infestation by varying populations of *Cinara tujafilina* del Guercio [82]. Data on the effect of intercropping on the content of TP indicate a possible increase in their content, which is considered beneficial due to their positive effect on human health and their role in enhancing resistance to certain plant diseases. Egyptian clover co-cultivated with *Cynara cardunculus* var. *altilis* increased the TP in *C. cardunculus* leaves. Intercropping barley and alfalfa in combination with the co-inoculation of arbuscular mycorrhizal fungi and plant growth-promoting rhizobacteria increased the TP and TF contents of barley grains by 132% and 343% [83]. Also, intercropping buckwheat and fenugreek in different proportions resulted in an increase in the TP and TF in buckwheat seeds by 13.6% (TP) and 11.3% and 22.9% (TF), respectively, in two consecutive years of study [25]. TP and TF in the roots of watermelon in intercropping with wheat was higher than in monocropping, and resistance to *Fusarium* wilt was also higher [84]. In our experiment, all protection measures reduced TF levels by about 24–77% depending on the treatment. This may be explained by the lower aphid colonization of broad bean plants in the treatments with companion plants and the chemically protected plots. Similarly to TP, foraging by aphids can increase flavonoids in the colonized plants [85].

## 4. Materials and Methods

### 4.1. Experimental Design

The field experiment was conducted at the Experimental Station of the University of Agriculture in Krakow Poland (50°06′45″ N 20°05′03″ E). The soil at the experimental site was degraded chernozem formed from loess, with the particle size distribution of loamy dust. The initial soil (before the experiment) showed a reaction close to neutral (pH in H_2_O = 6.06) and had a humus content of 2.28%. Broad beans of the Bizon variety were cultivated in companion planting with a mixture of CO, MU, and SA. The surface area of a single plot measured 36 m^2^ (6 m × 6 m). The space between broad bean rows was 65 cm, and companion plants were placed in the middle of the inter-rows. Companion plants were used in three different ratios. Oat served as a forecrop. The experiment included six treatments in three replications in line with the randomized block method.

The treatments were as follows:

1. broad beans in homogeneous cultivation without protection—control;
2. broad beans with a mixture of insectary plants in an equal proportion: 33% of MU, 33% of CO, and 33% of SA—mcs;
3. broad beans with a mixture of insectary plants in the proportion: 25% of MU, 25% of CO, and 50% of SA—mcSA;
4. broad beans with a mixture of insectary plants in the proportion: 25% of MU, 50% of CO, and 25% of SA—mCOs;
5. broad beans with a mixture of insectary plants in the proportion: 50% of MU, 25% of CO, and 25% of SA—MUcs;
6. broad beans in homogeneous cultivation protected with the use of synthetic pesticides (standard broad bean protection)—Ch.

The spacing between rows in the control and Ch was 50 cm, which is standard for broad bean cultivation. In the Ch treatment the following chemical insecticides were applied: Deltam AL (deltamethrin—0.0075 g) against pea leaf weevils when the first damage was noticed, Cyperkil Max 500 EC (cypermethrin—500 g) to combat the black bean aphid (BBCH 19–55, twice with 10-day interval), and Acelan 20 SP (acetamiprid—20%) to combat the broad bean weevil (*Bruchus rufimanus* Boh.) (BBCH 65–75). On the other hand, Mirador 250 SC (azoxystrobin—250 g/L (22.81%)), a fungicide against fungal diseases, was applied in the early flowering phase and repeated after 14 days.

Soil samples for soil parameters, element contents, and soil enzyme activity were collected from the surface layer (0–15 cm depth) from all treatments at the flowering stage of the broad bean. Each soil sample was collected with five sub-samples from each plot.

The assessment of basic soil parameters was performed in the Laboratory of Soil, Grounds, and Rock Analysis at the Institute of Earth Sciences, Faculty of Natural Sciences, University of Silesia. The particle size of the soil and pH values were determined as described previously [86,87]. The organic C and the total nitrogen content were estimated using the loss-on-ignition method [88] and the Kjeldahl method [89], respectively. Hydrolytic acidity was assessed using a modified version of Kappen’s method [90]. The plant material, for element contents in the broad bean, as well as for physiological parameters, was collected at the same time as soil samples. Fully developed, mature, and undamaged leaves of the broad bean were used. The leaves were collected from randomly selected plants from each plot at the flowering stage, which is considered the most suitable for measuring physiological response [91].

### 4.2. Elements Content in the Soil and Soil Enzyme Activity

The concentrations of heavy metals (Cd, Cu, Zn, Pb, Ni, Mn, and Fe) and macronutrients (K, Mg, Na, Ca, P, and S) in the soil were assessed using air-dried soil samples sieved through a 2 mm sieve, as described previously [92]. Metals and macronutrients were extracted from the soil samples using concentrated HNO_3_ (65%). For the HNO_3_-extractable fraction, 0.5 g of soil was placed in digestion tubes, soaked overnight in 5 mL of concentrated HNO_3_ at room temperature, and then further decomposed on an aluminum digestion block at 150 °C for 8 h, filtered, and diluted to 25 mL with deionized water. Metal concentrations in the filtered extracts were measured using inductively coupled plasma–atomic emission spectroscopy (SPECTROBLUE ICP-OES, Spectro Analytical Instruments, Kleve, Germany).

The soil enzyme activity was measured according to generally accepted methods, as follows: arylsulfatase [93,94] using potassium 4-nitrophenyl sulfate as substrate, 1 h of incubation, expressed in μg *p*-nitrophenol g^−1^ dry mass h^−1^; *β*-glucosidase [94,95] using salicin as substrate, 3 h of incubation, expressed in µg saligenin g^−1^ dry mass 3 h^−1^; dehydrogenase [96] using triphenyltetrazolium chloride as a substrate, 16 h of incubation, expressed in μg TPF(triphenylformazan) g^−1^ dm 16^−1^; acid phosphatase [96,97] using *p*-nitrophenolphosphate as a substrate, 1 h of incubation, expressed in µg *p*- nitrophenol g^−1^ dm h^−1^; FDA [98] using FDA as substrate, 2 h of incubation, expressed in µg fluorescein g^−1^ dm 2 h^−1^.

### 4.3. Element Contents in Broad Bean

To determine the concentrations of heavy metals (Cd, Cu, Zn, Pb, Ni, Mn, and Fe) and macroelements (K, Mg, Na, Ca, P, and S) in samples of plants, the plant material was initially washed with tap water to eliminate any substrate and dust, then rinsed twice with distilled water and dried at 105 °C. A 0.25 g portion of the dried plant material was placed into digestion vessels, treated with 4 mL of concentrated nitric acid, and pre-digested at room temperature for 24 h. Following this, the samples were heated in digital dry baths (The Labnet Digital Dry Baths, Spectra Services, Ontario, CA, USA), dual block; temperature range: +5 °C above ambient temperature to 150 °C; temperature uniformity: ±0.2 °C) at 120 °C until fully digested. After cooling to room temperature, the digested samples were filtered into 50 mL plastic bottles and diluted to 25 mL with deionized water [99]. Element concentrations were then measured using optical emission spectrometry with excitation by inductively coupled argon plasma (SPECTROBLUE ICP-OES, Spectro Analytical Instruments, Kleve, Germany).

### 4.4. Physiological Parameters in Broad Bean

The plant MDA content (indicative of the degree of peroxidation of the cytoplasm membrane under stress) was measured using the thiobarbituric acid method with a spectrophotometer (CE 1011, Cecil Instruments, Cambridge, England) at 532 nm [100]. Fresh leaf tissues (0.2 g) were homogenized using 2 mL of 5% trichloroacetic acid in an ice bath and centrifuged at 10,000 rpm for 10 min at 4 °C. About 2 mL of supernatant were mixed with 2 mL of 0.67% thiobarbituric acid and boiled for 30 min. After cooling, it was centrifuged. The absorption of the supernatant was carried out at 450, 532, and 600 nm, and then, the MDA content was expressed in micromoles per gram [100].

The ascorbic acid content of the leaf samples was determined using a spectrophotometer. The ascorbic acid content was calculated using the following formula [101]:

Ascorbic acid (mg × g^−1^ of fresh weight = [(E_0_ − E_s_ − E_t_)V/(W × 100] × 100 (1)

where V represents the volume of the extract, W denotes the weight of the leaf sample (in grams), and E_o_, E_s_, and E_t_ are the optical densities of the blank sample, the plant sample, and the sample with ascorbic acid, respectively.

The acid–ninhydrin method was employed to measure proline content, following the procedure previously outlined [102]. The plant material was homogenized in sulfosalicylic acid (3 g per 100 mL). The proline content, expressed as mM proline per gram of fresh weight, was calculated according to a method described previously [102].

The TP content, expressed as µg of chlorogenic acid per gram of fresh weight, was determined using the Folin–Ciocalteu method. To precipitate proteins, 250 mg of the leaf samples were boiled in 80% ethanol. The samples were then homogenized in 80% ethanol and centrifuged for 20 min at 4000× *g*. 0.2 mL of supernatant, 0.8 mL of distilled water, 0.5 mL of 20% Na_2_CO_3,_ and 0.125 mL of diluted Folin–Ciocalteu reagent were poured into test tubes. The reaction was incubated for 20 min, and then, the mixture was centrifuged for 20 min at 4000× *g* or filtered through a hard saucer. The absorbance of the solutions was measured at λ = 760 nm [103].

The spectrophotometric method was used to quantify TF, expressed as the quercetin equivalent, following the standard Christ–Müller procedure [104], as described for medicinal plants [105]. This method involves the acid hydrolysis of flavonol glycosides, followed by the formation of colored complexes of these flavonoid compounds with AlCl_3_. Absorbance was measured at a wavelength of 425.0 nm using a VIS spectrometer.

### 4.5. Growth Parameters of Broad Bean

Along with sampling for chemical and physiological analyses, broad bean plant growth parameters, such as length of the stem, number of stems per plant, number of composite leaves per stem, and mass of aboveground part of the plant, were evaluated on 15 randomly selected plants per plot.

### 4.6. Statistical Analysis

The data were pre-checked for normality (Shapiro–Wilk test with Lilliefors correction) and equality of variance (Levene’s test). The significance of differences between the mean values was tested by a one-way ANOVA (STATISTICA 13.0 software). Then a post hoc Tukey’s test was used at *p* ≤ 0.05. A PCA (STATISTICA 13.0 software) was used to assess the similarities and relations between the element levels and the enzymatic activity of the soil, the element levels and the physiological parameters of the broad bean, the growth parameters of the broad bean, and the different protection scenarios.

## 5. Conclusions

1. The introduction of insectary plant mixtures with different proportions of the individual species did not cause major changes in the content of elements in the soil with the exception of the mixture with 50% CO, which contributed to an increase in the HNO_3_-extractable fraction of K, Mg, Na, Ca, P, S, Cd, Cu, Ni, and Mn. The changes in the contents of these elements in broad bean leaves depended on the type of element and the proportion of individual components in the companion plant mixture. However, a general trend of increasing macronutrient content was observed, influenced by the presence of the companion plants. The insectary plant mixtures did not significantly affect the Pb, Ni, and Mn content of broad bean leaves, while the Fe content increased under their influence. Some of the tested mixtures also contributed to an increase in Zn, Cu, and Cd;
2. All types of companion plant mixtures used enhanced the activity of FDA with similar strength (by about 66–120%), while the mixture with 50% SA additionally caused an increase in arylsulfatase activity (more than 2-fold). There was no significant effect of companion plants on *β*-glucosidase, dehydrogenase, and acid phosphatase activities;
3. Companion plants improved the physiological condition of the protected plant, which was reflected in the reduced content of proline and TF. Proline content was most reduced in the leaves of the broad beans grown in association with a mixture with equal proportions of the individual components and with MU predominance (more than 6 fold in comparison to the control), while TF was reduced by all the proposed mixtures (by 24–77%), with the mixture with MU predominance being the least effective.

Considering the response of the soil and the protected plant to the neighborhood of the proposed mixtures and their effect on broad bean growth, it appears that the most suitable mixtures for use are those with an equal proportion of all three plant species or a mixture with a predominance of SA.

## Figures and Tables

**Table 1:** Contents (mg kg^−1^) of elements in experimental soil. Control—broad beans in homogeneous cultivation without protection; mcs—broad beans with a mixture of insectary plants in an equal proportion: 33% mustard, 33% coriander, and 33% sweet alyssum; mcSA—broad beans with a mixture of insectary plants in the proportion: 25% mustard, 25% coriander, and 50% sweet alyssum; mCOs—broad beans with a mixture of insectary plants in the proportion: 25% mustard, 50% coriander, and 25% sweet alyssum; MUcs—broad beans with a mixture of insectary plants in the proportion: 50% mustard, 25% coriander, and 25% sweet alyssum; Ch—broad beans in homogeneous cultivation protected with the use of synthetic pesticides (standard broad bean protection).

Treatment	K	Mg	Na	Ca	P	S	
control	2146.42 (±7.85) a *	1610.53 (±5.65) a	55.78 (±0.97) a	1981.73 (±48.04) ab	337.13 (±7.74) a	235.07 (±3.48) a	
mcs	2355.00 (±45.89) ab	1767.52 (±47.84) ab	57.92 (±0.14) ab	2013.82 (±15.14) ab	343.83 (±8.44) a	225.67 (±6.89) a	
mcSA	2190.38 (±6.71) a	1650.10 (±0.64) ab	58.50 (±0.46) ab	1980.88 (±32.55) ab	350.38 (±0.91) a	229.50 (±6.67) a	
mCOs	2553.37 (±13.93) b	1868.35 (±14.59) b	78.03 (±1.72) c	2368.35 (±12.12) c	459.23 (±2.65) c	266.57 (±7.05) b	
MUcs	2233.03 (±113.79) a	1709.38 (±103.68) ab	57.15 (±2.20) ab	1883.73 (±106.70) a	338.97 (±21.18) a	220.92 (±3.46) a	
Ch	2258.38 (±36.56) a	1681.78 (±35.44) ab	62.68 (±1.62) b	2151.83 (±34.98) bc	435.63 (±6.10) bc	248.27 (±4.46) ab	
	**Cd**	**Cu**	**Zn**	**Pb**	**Ni**	**Mn**	**Fe**
control	0.65 (±0.00) a	8.03 (±0.11) a	31.82 (±1.18) a	15.68 (±0.36) a	17.63 (±0.37) a	345.62 (±5.53) a	10,968.08 (±48.69) a
mcs	0.68 (±0.02) ab	8.15 (±0.10) a	31.73 (±0.54) a	17.12 (±0.54) ab	18.40 (±0.33) a	343.15 (±3.68) a	11,749.02 (±303.51) a
mcSA	0.63 (±0.01) a	7.98 (±0.13) a	31.75 (±0.43) a	19.08 (±1.40) ab	17.73 (±0.16) a	332.00 (±4.13) a	11,092.35 (±55.92) a
mCOs	0.80 (±0.00) b	11.52 (±0.72) b	57.43 (±15.89) a	19.45 (±0.50) ab	21.05 (±0.35) b	392.22 (±1.77) b	12,664.58 (±59.91) a
MUcs	0.67 (±0.06) ab	7.53 (±0.34) a	29.98 (±1.56) a	15.83 (±0.93) ab	17.83 (±0.97) a	319.70 (±17.07) a	11,348.98 (±772.63) a
Ch	0.72 (±0.02) a	8.52 (±0.07) a	35.65 (±0.89) a	20.88 (±1.89) b	18.07 (±0.53) a	347.32 (±5.04) a	11,323.77 (±227.73) a

* Values are shown as means ± SE. Different letters in columns indicate statistically significant differences (*p* ≤ 0.05, Tukey’s test).

**Table 2:** Contents (mg kg^−1^) of elements in broad bean. For description of treatments, see Table 1.

Treatment	K	Mg	Na	Ca	P	S	
control	18,264.75 (±827.19) a *	1440.93 (±207.57) a	226.97 (±17.34) a	9708.26 (±408.35) a	2035.68 (±224.34) a	1331.86 (±153.22) a	
mcs	26,424.00 (±658.30) b	2312.29 (±121.96) c	261.61 (±6.44) a	13,782.19 (±571.04) c	3342.05 (±158.69) b	2821.61 (±115.29) d	
mcSA	25,156.25 (±304.35) b	2193.39 (±95.45) bc	301.20 (±3.38) ab	13,822.51 (±21.26) c	2758.26 (±111.09) ab	2151.20 (±70.71) bc	
mCOs	27,180.25 (±347.60) b	1954.79 (±55.07) abc	486.46 (±7.26) d	13,546.00 (±244.41) c	2507.95 (±96.61) a	1897.82 (±68.83) b	
MUcs	31,070.23 (±1728.45) b	2419.20 (±130.50) c	409.89 (±6.87) cd	12,546.25 (±466.25) bc	3484.63 (±169.64) b	2627.35 (±118.59) cd	
Ch	18,023.43 (±2384.94) a	1566.29 (±147.17) ab	370.85 (±44.25) bc	10,707.39 (±967.98) ab	2115.92 (±181.30) a	1673.91 (±88.11) ab	
	**Cd**	**Cu**	**Zn**	**Pb**	**Ni**	**Mn**	**Fe**
control	n.d.	10.33 (±0.71) a	45.60 (±5.38) a	2.55 (±7.85) a	1.15 (±7.85) a	52.38 (±7.85) a	49.42 (±6.87) a
mcs	n.d.	12.37 (±0.57) ab	92.29 (±5.19) c	1.70 (±7.85) a	1.08 (±7.85) a	119.80 (±7.85) a	154.05 (±10.13) d
mcSA	0.12 (±0.00)	11.59 (±0.31) a	78.62 (±2.29) bc	5.06 (±7.85) a	7.60 (±7.85) a	102.81 (±7.85) a	105.01 (±1.95) bc
mCOs	n.d.	12.72 (±0.12) ab	59.81 (±2.50) ab	2.12 (±7.85) a	1.87 (±7.85) a	90.61 (±7.85) a	98.43 (±8.06) bc
MUcs	n.d.	15.05 (±0.71) b	85.06 (±4.97) c	2.91 (±7.85) a	2.33 (±7.85) a	106.22 (±7.85) a	131.87 (±8.06) cd
Ch	n.d.	9.44 (±1.30) a	57.26 (±3.52) a	2.10 (±7.85) a	2.76 (±7.85) a	66.71 (±7.85) a	74.98 (±5.85) ab

* Values are shown as means ± SE. Different letters in columns indicate statistically significant differences (*p* ≤ 0.05, Tukey’s test); n.d.—not detectable.

**Table 3:** Growth parameters of broad bean in years 2023–2024. For description of treatments, see Table 1.

Treatment	Length of Stem [cm]	No of Stems per Plant	No of Composite Leaves per Stem	Mass of Aboveground Part of Plant [g]
		2023		
control	60.37 (±1.55) a *	2.48 (±0.17) a	11.22 (±0.64) a	260.48 (±25.43) a
mcs	81.08 (±2.50) cd	3.03 (±0.07) b	11.67 (±0.17) a	344.45 (±11.37) b
mcSA	78.23 (±1.86) cd	3.07 (±0.14) b	11.62 (±0.18) a	324.73 (±14.65) ab
mCOs	74.99 (±1.36) bc	2.87 (±0.08) ab	11.49 (±0.15) a	275.43 (±7.72) ab
MUcs	84.08 (±2.95) d	2.83 (±0.10) ab	11.14 (±0.16) a	294.77 (±24.25) ab
Ch	69.54 (±1.44) b	3.21 (±0.16) b	12.47 (±0.30) a	437.69 (±22.60) c
		2024		
control	34.74 (±1.53) a	2.76 (±0.20) a	10.98 (±0.50) a	88.36 (±8.88) a
mcs	38.18 (±1.17) a	2.83 (±0.17) a	13.22 (±0.84) ab	107.48 (±10.54) ab
mcSA	37.21 (±1.66) a	4.28 (±0.30) b	13.04 (±0.76) ab	146.71 (±12.08) b
mCOs	39.12 (±1.17) a	3.20 (±0.26) a	13.33 (±0.70) ab	116.57 (±9.93) ab
MUcs	40.43 (±1.36) a	2.83 (±0.24) a	13.30 (±0.55) ab	108.28 (±9.88) ab
Ch	46.40 (±1.53) b	3.13 (±0.14) a	15.41 (±0.44) b	121.66 (±11.47) ab

* Values are shown as means ± SE. Different letters in columns indicate statistically significant differences (*p* ≤ 0.05, Tukey’s test).

## Data Availability

Data are contained within the article and Supplementary Materials.

## Supplementary Materials

The following supporting information can be downloaded at https://www.mdpi.com/article/10.3390/molecules29246031/s1: Table S1: Parameters of the soil taken from the plots where broad bean (used as main protected plant) grown in the company of a mixture of three insectary plant species: sweet alyssum, coriander, and white mustard, at different shares of the individual components in the mixture (mcs, mcSA, mCOs, MUcs) as well as in homogeneous cultivation with (Ch) or without protection (control); Table S2: Granulometric composition [% of specific fraction in mm] of the soil taken from the plots where broad bean (used as main protected plant) grown in the company of a mixture of three insectary plant species: sweet alyssum, coriander, and white mustard, at different shares of the individual components in the mixture (mcs, mcSA, mCOs, MUcs) as well as in homogeneous cultivation with (Ch) or without protection (control); Table S3: Results of the statistical analysis (one-way ANOVA) on the content of elements in the soil taken from the plots where broad bean (used as main protected plant) grown in the company of a mixture of three insectary plant species: sweet alyssum, coriander, and white mustard, at different shares of the individual components in the mixture (mcs, mcSA, mCOs, MUcs) as well as in homogeneous cultivation with (Ch) or without protection (control); Table S4: Results of the statistical analysis (one-way ANOVA) on soil enzyme activity levels. Soil samples taken from the plots where broad bean (used as main protected plant) grown in the company of a mixture of three insectary plant species: sweet alyssum, coriander, and white mustard, at different shares of the individual components in the mixture (mcs, mcSA, mCOs, MUcs) as well as in homogeneous cultivation with (Ch) or without protection (control); Table S5: Results of the statistical analysis (one-way ANOVA) on the content of elements in broad bean (used as main protected plant) grown in the company of a mixture of three insectary plant species: sweet alyssum, coriander, and white mustard, at different shares of the individual components in the mixture (mcs, mcSA, mCOs, MUcs) as well as in homogeneous cultivation with (Ch) or without protection (control); Table S6: Results of the statistical analysis (one-way ANOVA) on the physiological parameters of broad bean (used as main protected plant) grown in the company of a mixture of three insectary plant species: sweet alyssum, coriander, and white mustard, at different shares of the individual components in the mixture (mcs, mcSA, mCOs, MUcs) as well as in homogeneous cultivation with (Ch) or without protection (control); Table S7: Results of the statistical analysis (one-way ANOVA) on the growth parameters of broad bean (used as main protected plant) grown in the company of a mixture of three insectary plant species: sweet alyssum, coriander, and white mustard, at different shares of the individual components in the mixture (mcs, mcSA, mCOs, MUcs) as well as in homogeneous cultivation with (Ch) or without protection (control).
