# Supplementary material for: Effects of Intercropped Insectary Plants (Sweet Alyssum, Coriander, and White Mustard) on Elemental Composition and Antioxidant Levels in Broad Bean Plants
Source: Molecules. 2024 Dec 21;29(24):6031. doi: 10.3390/molecules29246031 (PMC11678412; doi:10.3390/molecules29246031)
Supplement: Supplementary file 1 [file molecules-29-06031-s001.zip › molecules-3341575-supplementary.pdf]

Table S1. Parameters of the soil taken from the plots where broad bean (used as main protected plant) grown in the company of a mixture of three insectary plant species: sweet alyssum, coriander, and white mustard, at different shares of the individual components in the mixture (mcs, mcSA, mCOs, MUcs) as well as in homogeneous cultivation with (Ch) or without protection (control).

**Treatment**	**pH (H_2O)**	**pH (KCl)**	**$C_{org.}$ (%)**	**N_T (%)**	**Hh (cmol/kg)**
control*	5.92	4.81	3.76	0.093	5.56
mcs	6.05	4.96	2.42	0.166	6.12
mcSA	5.92	4.79	0.99	0.064	5.28
mCOs	5.94	4.93	4.19	0.117	5.80
MUcs	5.84	4.67	4.63	0.124	5.80
Ch	6.03	4.85	3.78	0.078	5.36

* control - broad beans in homogeneous cultivation without protection; mcs - broad beans with a mixture of insectary plants in an equal share: 33% of mustard, 33% of coriander, 33% of sweet alyssum; mcSA - broad beans with a mixture of insectary plants in the proportion: 25% of mustard, 25% of coriander, 50% of sweet alyssum; mCOs - broad beans with a mixture of insectary plants in the proportion: 25% of mustard, 50% of coriander, 25% of sweet alyssum; MUcs -broad beans with a mixture of insectary plants in the proportion: 50% of mustard, 25% of coriander, 25% of sweet alyssum; Ch - broad beans in homogeneous cultivation protected with the use of synthetic pesticides (standard broad bean protection); C_{org} – organic carbon; N_T – total nitrogen content; Hh - hydrolytic acidity.

Table S2. Granulometric composition [% of specific fraction in mm] of the soil taken from the plots where broad bean (used as main protected plant) grown in the company of a mixture of three insectary plant species: sweet alyssum, coriander, and white mustard, at different shares of the individual components in the mixture (mcs, mcSA, mCOs, MUcs) as well as in homogeneous cultivation with (Ch) or without protection (control). For treatments description see Table S1.

**Treatment**	>0.25	0.25-0.1	0.1-0.05	0.05-0.02	0.02-0.006	0.006-0.002	<0.002
control	-	3	7	44	24	9	13
mcs	-	3	9	43	23	9	13
mcSA	-	4	8	44	23	8	13
mCOs	-	1	10	42	23	9	15
MUcs	-	4	9	43	22	8	14
Ch	-	2	8	43	24	8	15

Table S3. Results of the statistical analysis (one-way ANOVA) on the content of elements in the soil taken from the plots where broad bean (used as main protected plant) grown in the company of a mixture of three insectary plant species: sweet alyssum, coriander, and white mustard, at different shares of the individual components in the mixture (mcs, mcSA, mCOs, MUcs) as well as in homogeneous cultivation with (Ch) or without protection (control).

Elements	Sum of Squares	df	Mean Square	F	p
K	325114	5	65023	7.791	0.001785
Mg	127601	5	25520	3.510	0.034685
Na	1044	5	209	35.963	0.000001
Ca	447100	5	89420	10.938	0.000387
P	45130	5	9026	28.882	0.000003
S	4337	5	867	9.345	0.000800
Fe	5752953	5	1150591	3.068	0.051788
Cd	0	5	0	5.273	0.008606
Cu	32	5	6	18.766	0.000027
Zn	1646	5	329	2.556	0.084918
Pb	67	5	13	3.814	0.026673
Ni	25	5	5	6.348	0.004186
Mn	9093	5	1819	9.542	0.000728

Table S4. Results of the statistical analysis (one-way ANOVA) on soil enzyme activity levels. Soil samples taken from the plots where broad bean (used as main protected plant) grown in the company of a mixture of three insectary plant species: sweet alyssum, coriander, and white mustard, at different shares of the individual components in the mixture (mcs, mcSA, mCOs, MUcs) as well as in homogeneous cultivation with (Ch) or without protection (control).

Enzymes	Sum of Squares	df	Mean Square	F	p
Arylsulphatase	1029	5	206	22.266	0.000011
β-glucosidase	16289878	5	3257976	37.196	0.000001
Dehydrogenase	1	5	0	4.022	0.022431
FDA*	138	5	28	11.745	0.000276
Acid phosphatase	92362	5	18472	5.718	0.006320

* FDA (fluorescein diacetate)

Table S5. Results of the statistical analysis (one-way ANOVA) on the content of elements in broad bean (used as main protected plant) grown in the company of a mixture of three insectary plant species: sweet alyssum, coriander, and white mustard, at different shares of the individual components in the mixture (mcs, mcSA, mCOs, MUcs) as well as in homogeneous cultivation with (Ch) or without protection (control).

Elements	Sum of Squares	df	Mean Square	F	p
K	405536738	5	81107348	16.211	0.000057
Mg	2433704	5	486741	8.948	0.000972
Na	142997	5	28599	23.713	0.000008
Ca	46095893	5	9219179	10.799	0.000411
P	5550865	5	1110173	13.979	0.000119
S	4837065	5	967413	28.315	0.000003
Fe	21353	5	4271	26.888	0.000004
Cd	0	5	0	36204289.00	0.000000
Cu	58	5	12	7.458	0.002150

Zn	4978	5	996	19.061	0.000025
Pb	22	5	4	4.216	0.019138
Ni	90	5	18	33.604	0.000001
Mn	9819	5	1964	21.675	0.000013

Table S6. Results of the statistical analysis (one-way ANOVA) on the physiological parameters of broad bean (used as main protected plant) grown in the company of a mixture of three insectary plant species: sweet alyssum, coriander, and white mustard, at different shares of the individual components in the mixture (mcs, mcSA, mCOs, MUcs) as well as in homogeneous cultivation with (Ch) or without protection (control).

Parameter	Sum of Squares	df	Mean Square	F	p
MDA*	0.065686	5	0.013137	1.764	0.194817
Ascorbic acid	0.001452	5	0.000290	1.063	0.426934
Proline	342.0386	5	68.40773	70.437	0.000000
TP	367.7321	5	73.54641	4.021	0.022434
TF	92597.93	5	18519.59	21.535	0.000013

*MDA – malondialdehyde content; TP - total phenolic content; TF - total flavonoids content

Table S7. Results of the statistical analysis (one-way ANOVA) on the growth parameters of broad bean (used as main protected plant) grown in the company of a mixture of three insectary plant species: sweet alyssum, coriander, and white mustard, at different shares of the individual components in the mixture (mcs, mcSA, mCOs, MUcs) as well as in homogeneous cultivation with (Ch) or without protection (control).

Parameter	Sum of Squares	df	Mean Square	F	p
	2023				
Length of stem [cm]	11420.87	5	2284.17	18.266	0.000000
No of stems per plant	9.62	5	1.92	4.157	0.001358
No of composite leaves/stem	33.49	5	6.70	2.097	0.067901
Mass of aboveground part of plant [g]	608602.98	5	121720.60	11.289	0.000000
	2024				
Length of stem [cm]	2323.18	5	464.64	7.799	0.000001
No of stems per plant	47.24	5	9.45	6.355	0.000019
No of composite leaves/stem	291.67	5	58.33	4.655	0.000518
Mass of aboveground part of plant [g]	55197.18	5	11039.44	3.334	0.006702
